# Supplementary material for: Splice-Junction-Based Mapping of Alternative Isoforms in the Human Proteome
Source: Cell Rep. Author manuscript; Available in PMC 2020 Jan 15. (PMC6961840; doi:10.1016/j.celrep.2019.11.026)
Supplement: 2 [file NIHMS1546469-supplement-2.zip › DF1/PXD010154/Thyroid/Thyroid_28_TG_SECYMEITALLASICWVSANIFEYQVDAQPLRPCELQR.pdf]

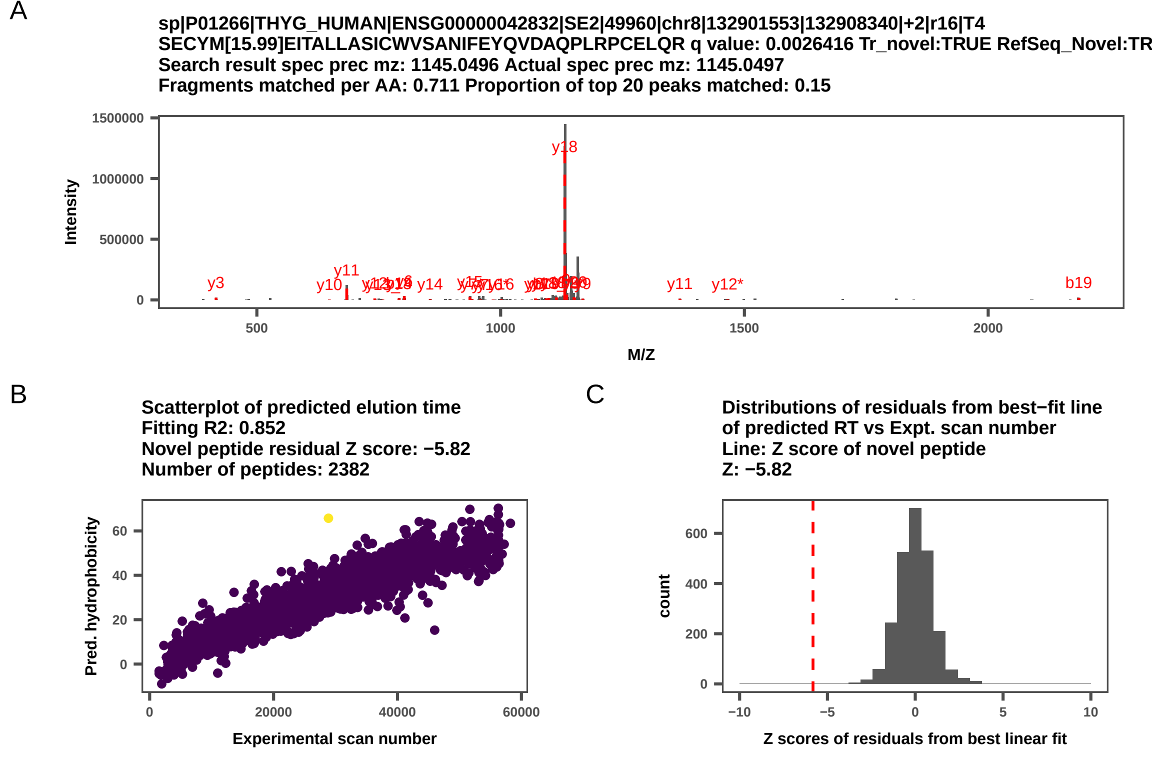
A
sp|P01266|THYG_HUMAN|ENSG00000042832|SE2|49960|chr8|132901553|132908340|+2|r16|T4
SECYM[15.99]EITALLASICWVSANIFEYQVDAQPLRPCELQR q value: 0.0026416 Tr_novel:TRUE RefSeq_Novel:TR
Search result spec prec mz: 1145.0496 Actual spec prec mz: 1145.0497
Fragments matched per AA: 0.711 Proportion of top 20 peaks matched: 0.15
Intensity
1500000
1000000
500000
0
y18
y3
y11
y10
y14
y11
y12*
b19
500
1000
1500
2000
M/Z
B
Scatterplot of predicted elution time
Fitting R2: 0.852
Novel peptide residual Z score: −5.82
Number of peptides: 2382
Pred. hydrophobicity
60
40
20
0
0
20000
40000
60000
Experimental scan number
C
Distributions of residuals from best−fit line
of predicted RT vs Expt. scan number
Line: Z score of novel peptide
Z: −5.82
count
600
400
200
0
−10
−5
0
5
10
Z scores of residuals from best linear fit
